# The moderating role of co-occurring attention-deficit hyperactivity disorder in social skills group training for autistic children and adolescents

**DOI:** 10.1177/13623613251331993

**Published:** 2025-04-23

**Authors:** Anna Fridell, Nora Choque Olsson, Christina Coco, Sven Bölte, Ulf Jonsson

**Affiliations:** 1 Center of Neurodevelopmental Disorders (KIND), Centre for Psychiatry Research; Department of Women’s and Children’s Health, Karolinska Institutet & Stockholm Health Care Services, Region Stockholm, Stockholm, Sweden; 2 Child and Adolescent Psychiatry, Stockholm Health Care Services, Region Stockholm, Stockholm, Sweden; 3 Department of Psychology, Stockholm University, Stockholm, Sweden; 4 Curtin Autism Research Group, Curtin School of Allied Health, Curtin University, Perth, Australia; 5 Department of Medical Sciences, Child and Adolescent Psychiatry, Uppsala University, Uppsala, Sweden

**Keywords:** attention-deficit hyperactivity disorder, autism, clinically relevant change, interaction, intervention, moderator analysis, personalized medicine, reliable change, response

## Abstract

Social skills group training is frequently used to support autistic children and adolescents in coping with everyday social demands and expectations. Meta-analytic studies suggest small to medium effect sizes. Effect moderators must be better understood to inform implementation and allow optimization of intervention protocols. Using data from two pragmatic randomized trials (*N* = 241) of the KONTAKT™ social skills group training program as an add-on to standard care in clinical settings, we aimed to explore the moderating role of co-occurring attention-deficit hyperactivity disorder in children (7–12 years) and adolescents (13–18 years). Two distinct response criteria were defined based on the parent-rated Social Responsiveness Scale: reliable improvement (⩾25 points) and clinically relevant improvement (⩾10 points). Moderator analyses indicated that the intervention effect was moderated by co-occurring attention-deficit hyperactivity disorder and age group. Logistic regressions stratified by co-occurring attention-deficit hyperactivity disorder showed significant effects among those without attention-deficit hyperactivity disorder (*n* = 63), both in terms of reliable improvement (odds ratio: 11.85, *p* = 0.002) and clinically relevant improvement (odds ratio: 10.00, *p* = 0.001). In contrast, no significant effects were observed in those with co-occurring attention-deficit hyperactivity disorder (*n* = 178). Explorative analyses further stratified by age suggested significant effects in adolescents, but not children, with co-occurring attention-deficit hyperactivity disorder. The findings preliminarily suggest that autistic children with attention-deficit hyperactivity disorder may need other or additional support.

**Lay abstract:**

Social skills group training can help some autistic children and adolescents improve their social life. Still, the positive effects may be less clear for those who also have attention-deficit hyperactivity disorder. We used data from two previous projects evaluating the effects of a social skills group training program called KONTAKT™ as an addition to the common healthcare provided. Our study included 241 children (8–12 years) and adolescents (13–18 years). To determine whether the participants had improved their social skills, we used ratings provided by the parents before and after the training and 3 months later. We then explored if KONTAKT led to improvement for autistic children and adolescents with and without attention-deficit hyperactivity disorder. Autistic children and adolescents often struggle to understand others and express themselves in everyday social situations. These challenges can create barriers to well-being and future life chances. Social skills group training can improve social skills in some autistic youths, but not all will benefit equally from the training. It is therefore important to better understand whether some groups need more support or other forms of assistance. Many autistic children and adolescents also have attention-deficit hyperactivity disorder, which can make the training of social skills more complicated. We found that both children and adolescents can benefit from KONTAKT. Still, preadolescent autistic children with attention-deficit hyperactivity disorder did not seem to improve as a result of the training. Based on these findings, it is important to find additional strategies to support this specific group of autistic children in handling social situations.

## Introduction

Social skills group training (SSGT) is a frequently used intervention to support verbal autistic children and adolescents within the average intellectual range to cope with everyday social demands and expectations. Typically conducted in small groups, this training may incorporate antecedent-based strategies, reinforcement, and behavioral practice techniques. It also includes instructions on pre-determined social skills, as well as more naturalistic and performance-based methods (Afsharnejad et al., 2024; Hall et al., 2018).

The primary aim of SSGT is to develop social skills and understanding (social competency). In addition, secondary outcome measures in the research reflect that the intervention also intends to promote positive psychosocial outcomes in a broader sense, including peer inclusion, well-being, and quality of life (Moody et al., 2022). In the long term, self-efficacy in social situations, along with strategies to reduce contextual barriers and enhance the person–environment fit (Lai et al., 2020), may potentially mitigate the risk of co-occurring mental health conditions (Demir et al., 2012; Stimpson et al., 2021), loneliness, bullying (Hymas et al., 2024; Locke et al., 2010; Maiano et al., 2016; Zeedyk et al., 2014), academic underachievement, and school absenteeism (Anderson, 2020; Leifler et al., 2022; Nordin et al., 2023).

Meta-analytic studies suggest SSGT improves social competency in autistic children and adolescents (Wolstencroft et al., 2018), mainly by increasing social knowledge (Gates et al., 2017). While small to medium effect sizes have been reported on a group level (Gates et al., 2017), some results indicate that a substantial proportion of participants show no or little benefit from the intervention (e.g. Dekker et al., 2021; White et al., 2013). Similarly, randomized trials of KONTAKT™, a manualized SSGT program, suggest that females (as opposed to males) and adolescents (as opposed to children) benefit more from the training (Choque Olsson et al., 2017). It has also been reported that genetics may affect the outcome of KONTAKT (Li et al., 2020, 2022; Tammimies et al., 2019). Indeed, individual differences in outcome should be expected, given the heterogeneity of the target population (Kirby et al., 2016; Lord et al., 2022). Better insight into individual response patterns could help improve precision in clinical decision-making (Lord et al., 2022), prevent unnecessary or harmful clinical action, and mitigate service-supply shortages (Bölte et al., 2020; Malik-Soni et al., 2022).

The clinical complexity of co-occurring attention-deficit hyperactivity disorder (ADHD) and autism has been emphasized in previous research, for instance, concerning response to pharmacological treatment (Rodrigues et al., 2021). The prevalence of co-occurring ADHD in autism has been estimated to 28% (Lai et al., 2019), with substantially higher prevalence rates reported in some studies (Hossain et al., 2020). Social functioning has been reported to differ between autistic children and adolescents with and without ADHD (Harkins et al., 2022; Piltz et al., 2023). Youth with ADHD may be more likely to struggle with enacting social skills rather than knowing what skills to apply (Mikami et al., 2017). This is likely linked to core symptoms, including inattention, impulsivity, and hyperactivity (World Health Organization, 2019/2021). Moreover, functional challenges stemming from the high rates of co-occurring mental health conditions in this population (Casseus et al., 2023) can aggravate the situation further, affecting readiness for change, intervention adherence, engagement, and outcome.

Notably, children and adolescents with ADHD alone may not experience similar gains from SSGT as their autistic counterparts (Gates et al., 2017; Storebø et al., 2019). The research to date on co-occurring ADHD and the outcome of SSGT remains inconclusive. For instance, some studies have found that those with co-occurring ADHD improve less in social competency than those with an autism diagnosis alone (Antshel et al., 2011; Gerber et al., 2022). Conversely, a recent study of the PEERS social skills training program found similar changes in social competency in children and adolescents with ADHD, autism, or a combination of both (Geannopoulos et al., 2024). Furthermore, it is not clear that pre-existing differences in social competency impact the outcome. Harkins and Mazurek (2024) observed that co-occurring ADHD did not affect the outcome of a group-based Social Competence Intervention negatively, even though autistic children with ADHD had lower social awareness at baseline than children with autism alone. Similarly, Dekker et al. (2021) observed that although autistic children with ADHD had greater baseline social challenges, these differences did not seem to impact their improvement in social competency following SSGT.

An important shortcoming of previous studies is the lack of a randomized design, especially since moderator effects should preferably be tested in randomized controlled trials (RCTs) (Kraemer et al., 2002). In this study, we sought to add to the extant literature by exploring the moderating effect of co-occurring ADHD on the outcome of KONTAKT for autistic children and adolescents. We utilized a combined clinical sample from two RCTs evaluating KONTAKT as an add-on to standard care in child and adolescent mental health services compared to standard care alone. We used two distinct response criteria (reliable improvement and clinically relevant improvement) to investigate individual-level outcomes. Since separate skills training groups were conducted for children (7–12 years) and for adolescents (13–18 years), the moderating effect of co-occurring ADHD was explored further by stratifying by age group. To enhance the clinical relevance of the findings, we calculated the numbers needed to treat (NNT) and adjusted odds ratios (aOR) for the response criteria. The study had the following objectives:

1. To investigate whether individual response to KONTAKT is moderated by co-occurring ADHD.
2. To further explore the potential moderator effect of co-occurring ADHD in children (age 7–12) and adolescents (age 13–18).

## Methods

### Study design

This study used data from two parallel-group, multi-center, pragmatic RCTs investigating the effectiveness of 12 sessions (*N* = 296; Choque Olsson et al., 2017) and 24 sessions (*N* = 50; Jonsson et al., 2019) of KONTAKT as an add-on to standard care (henceforth KONTAKT) as compared with standard care alone in child and adolescent mental health service settings. The RCTs were conducted at 13 child and adolescent mental health service units between August 2012 and October 2015 and coordinated at the Center of Neurodevelopmental Disorders at Karolinska Institute (KIND). The study design and procedures of the two trials were identical in recruitment strategies, staff training, supervision, intervention implementation, criteria for inclusion and exclusion of participants, and outcome measures. Block randomization in a 1:1 ratio, stratified by age groups (children aged 7–12 years and adolescents aged 13–18 years), was conducted by a senior researcher using computer-generated numbers (http://www.random.org/). Outcome assessment occurred at baseline, postintervention, and 3-month follow-up. Additional details are available in the original publications (Choque Olsson et al., 2017; Jonsson et al., 2019).

The original RCTs as well as the present analyses were approved by the Regional Ethical Review Board [2012/385-31/4; 201441124-32; 201442189-32; 2019 02348] and were pre-registered with ClinicalTrials.gov [NCT01854346].

### Recruitment and participants

Eligible participants were children (8–12 years) and adolescents (13–17 years) meeting the International Classification of Diseases, 10th Revision (ICD-10; World Health Organization, 2019/2021) criteria for a diagnosis on the autism spectrum (F84.0, F84.1, F84.5, or F84.9), ascertained by multidisciplinary assessment teams in regular healthcare services. Diagnostic criteria were corroborated by cutoff scores (modules 3 and 4) on the Autism Diagnostic Observation Schedule (ADOS; Lord et al., 2000) or ADOS-2 (Lord et al., 2014) conducted by certified mental health professionals. Unless the individual presents with co-occurring mental healthcare needs or requires pharmacological treatment, autism is typically managed in habilitation services in Sweden, a provider specialized in disability support. Since this study was conducted within the child and adolescent mental health service, all participants presented with one or more co-occurring conditions. Eligible participants had a co-occurring diagnosis of ADHD (F90.0 or F90.8), anxiety disorder (F40, F41, or F43), or depression (F32 or F33) ascertained by multidisciplinary assessment teams. Diagnoses were obtained from medical records. If new information about depression and anxiety surfaced during the intake interview, or if it was unclear whether a previously diagnosed depression or anxiety disorder was still ongoing, this was corroborated by the research team using the Kiddie-Schedule of Affective Disorders and Schizophrenia (K-SADS; Kaufman et al., 1997). All participants had a full-scale IQ > 70, determined by the Wechsler Intelligence Scale for Children—third or fourth edition (Wechsler, 1991, 2004), and sufficient proficiency in Swedish.

### Sample characteristics

**Figure 1.**
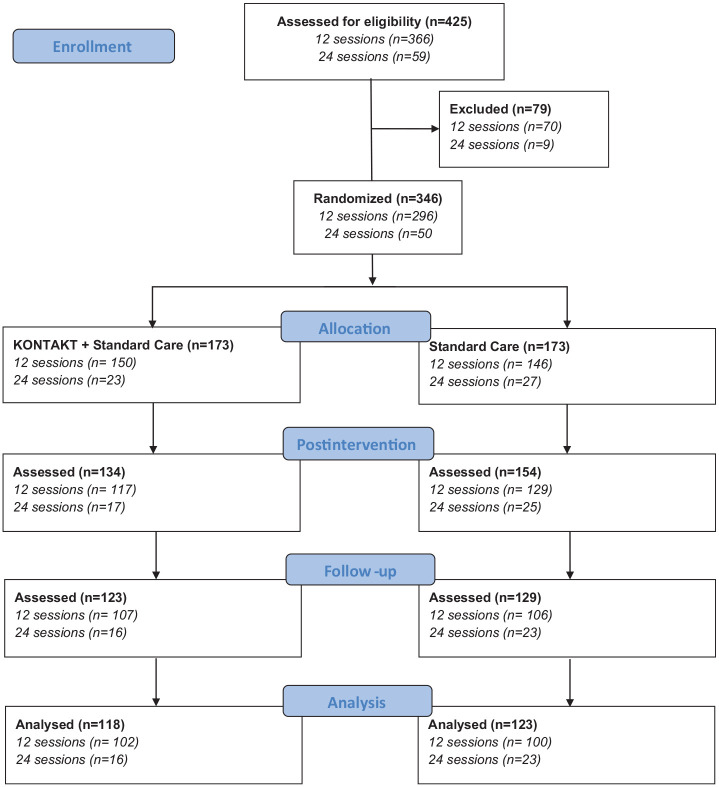
Flow diagram of samples from two randomized trials evaluating 12 and 24 weeks of social skills training (KONTAKT) as an add-on to standard care for autistic children and adolescents.

The analytic sample for this study included participants with complete data from all three assessment points, comprising 241 (70%) of the 346 original participants (see Figure 1 and Supplementary Material, Table S1).

Clinical and demographic characteristics were distributed evenly among completers and non-completers, with the exception that non-completers were more likely to take sleep-inducing medication, have a full-scale IQ and working memory subindex below 85, and score higher at preintervention on the parent-rated Social Responsiveness Scale (SRS; see Supplementary Material, Table S2).

A total of 130 children and 111 adolescents were included, of which 178 had co-occurring ADHD and 63 did not. Co-occurring ADHD was less common among adolescents (65.8%) than among children (80.8%). The subgroup without ADHD had diagnoses of depression (23.8%), anxiety disorder (60.3%), or both (15.9%). These conditions were significantly less common among those with co-occurring ADHD (*p* < 0.001). The distribution of most baseline characteristics was similar in children and adolescents with and without ADHD. However, the sub-sample with ADHD had lower age at baseline (*p* = 0.005), lower parental age (*p* = 0.003), and lower full-scale IQ (*p* = 0.046) and working memory index (*p* = 0.001). They were also more likely to receive central nervous system stimulants (*p* < 0.001) and sleep-inducing medication (*p* = 0.031), and less likely to receive cognitive behavioral therapy (*p* = 0.042). When stratified by age group, the differences in working memory and central nervous system stimulant use persisted among children and adolescents. The likelihood of receiving cognitive behavioral therapy (CBT) only differed among the adolescents (Supplementary Material, Table S1).

### Intervention

KONTAKT is a manualized SSGT program for autistic children and adolescents within the average intellectual range (Bölte, 2018). It aims to improve social interaction and communication skills, social motivation, awareness of self and others, problem-solving capacities, and self-confidence. The program applies strategies from cognitive behavioral therapy and learning principles (including psychoeducation, behavioral activation, and observational learning) through various mandatory, recurring, and variable intervention activities. Intervention activities include individual goal identification, group discussion, role-play, emotion-processing training, group activities, and homework. One session per week is scheduled for 12 or 24 weeks. Parents participate in three (12-session version) or six (24-session version) parallel sessions to support their child’s program participation. While the first 12 sessions are equivalent in both versions, the remaining sessions of the 24-session version are structured around the needs of individual groups. Specifically, group trainers will tailor themes for group discussions, group activities, and homework to individual needs.

Groups are led by two trainers and include four to eight participants, with separate groups for children and adolescents. The sessions are shorter for children (60 min) than adolescents (90 min), but the overall content is similar for both age groups. Additional support is provided based on clinical assessment and individual needs, including opportunities for breaks, movement, and cognitive supports (for further details; Choque Olsson et al., 2017; Jonsson et al., 2019). KONTAKT is currently available in services for autistic children and adolescents in several countries (Australian; Girdler et al., 2021; German; Herbrecht et al., 2007; Norwegian; Larsen, 2021).

### Standard care

Standard care included ongoing support or treatment in regular child and adolescent mental health services (e.g. pharmacological treatments, occupational therapy, psychoeducation, general counseling, CBT, cognitive assistive technologies, and weighted blankets).

### Study variables

#### Moderating variable

The moderating variable of primary interest in this study was co-occurring ADHD, defined as a clinical diagnosis of ADHD according to the ICD-10 criteria. Age group, dichotomized into children (8–12 years of age) and adolescents (13–18 years of age), was also explored as a moderator due to its central role in how the intervention and study were designed.

#### Outcome variables

The raw scores of the primary outcome, the parent-rated SRS (Constantino & Gruber, 2005, 2012), were dichotomized into responders and non-responders according to two distinct criteria: reliable improvement and clinically relevant improvement (see definitions below). SRS has commonly been used as the primary outcome in trials evaluating the effects of SSGT (Moody et al., 2022). The SRS is a 65-item instrument measuring autistic-like traits across five domains—social awareness, social cognition, social communication, social motivation, and autistic mannerisms. The Swedish version has demonstrated high external validity, excellent internal consistency (Cronbach’s α = 0.91–0.97), and good test–retest reliability of 0.84–0.97 (Bölte et al., 2008).

Reliable improvement was based on the reliable change index (RCI; Jacobson & Truax, 1991) and was calculated in two steps. First, the adjusted standard error of measurement of the difference (*S_Diff_*) was obtained using the following formula, where SD is the standard deviation, and r refers to the test–retest reliability

$$SDiff=SD*\sqrt{2}*\sqrt{1-r}$$

The Swedish clinical norm population estimated parameter of test–retest reliability (*r* = 0.87) was obtained from the Swedish SRS-2 manual (Constantino & Gruber, 2019). The standard deviation was based on the preintervention parent-rated SRS scores of the combined samples of the two KONTAKT trials (SD = 24.72).

Second, the RCI was calculated using the following formula

RCI=SDiff*1.96

With an RCI of 24.71, reliable improvement was defined as a positive change of ⩾25 points on the SRS, either from pre- to postintervention or from preintervention to 3-month follow-up.

The clinically relevant change was defined as ⩾10 points, based on the recommendation by the authors of the SRS (Bölte et al., 2008; Constantino & Gruber, 2005, 2012, 2019; personal communication C. Constantino, 2008). To increase the certainty of the results, the clinically relevant improvement required ⩾10 points improvement from pre- to postintervention and a maintained improvement of ⩾10 points at the 3-month follow-up.

#### Covariates

Relevant covariates were selected from available background information, based on theoretical assumptions, baseline differences between the compared groups, and previous results from the two trials (Choque Olsson et al., 2017; Jonsson et al., 2019). Parent-rated SRS raw scores at preintervention were included as a continuous variable to control for baseline differences. Chronological age (at baseline) in years was included as a continuous variable. Sex assigned at birth (female/male), intervention length (12 weeks/24 weeks), full-scale IQ scores dichotomized at one standard deviation below average (IQ < 85), depression, anxiety disorder, and use of central nervous system stimulants were dichotomous variables. Due to small numbers treated with CBT, this variable could not be used. For the same reason, sleep medication (yes/no) could only be entered in models including the participants with co-occurring ADHD (see below under Sensitivity analyses). Parental age was not included as it was deemed of limited relevance for the analyses, while the working memory sub-scale was deemed superfluous when adjusting for full-scale IQ.

### Statistical analysis

#### Main analysis

Baseline characteristics of participants with and without co-occurring ADHD were compared in the total sample and stratified by age group, using independent *t*-tests for continuous variables and chi-square tests for categorical variables. The same strategy was used to analyze the attrition.

We investigated the moderating effects of ADHD in binary logistic regression models. First, the three-way interaction term of ADHD (yes or no) by study arm (KONTAKT or standard care) by age group (children or adolescents) was entered, together with all underlying two-way interactions and main effects. Second, the two-way interactions and their underlying main effects were tested separately. Exploratively, the two-way interactions were analyzed further after the sample had been stratified by co-occurrence of ADHD (age group by study arm) and age group (ADHD by study arm). Identical analytic strategies were used to analyze reliable improvement and clinically relevant improvement.

To estimate the effect of KONTAKT (compared to standard care), unadjusted odds ratios and aOR for both response criteria were calculated using univariate and multivariate logistic regression modeling. This was done in (a) the total sample, (b) stratified by presence of ADHD, and (c) stratified and age group. Models were adjusted for sex, age at baseline, depression, anxiety disorder, central nervous system stimulants, intervention length, and baseline SRS. In a final explorative step, children and adolescents were further stratified by co-occurring ADHD. Due to the small samples of these stratified analyses, adjustments were limited to sex, age at baseline, and baseline SRS.

NNT signify the number of participants who need to receive the intervention (compared to standard care only) for one additional participant to reach the intended outcome. The following formula was used, where EER was the event rate (i.e. number of responding participants divided by total number of participants) for the experimental group (i.e. KONTAKT), and CER the event rate for the control group (i.e. standard care)

$$NNT=\frac{1}{EER-CER}$$

A low NNT indicates that the intervention is effective compared to the control condition. A negative NNT demonstrates that the control condition is more beneficial than the interventions. NNTs were rounded up to the nearest whole number.

In accordance with the per-protocol principle, the main analyses include participants without missing data on the outcome variable. Throughout, a 5% two-tailed test of significance was used. No correction for multiple testing was done. All analyses were conducted using IBM SPSS Statistics 28.0.

#### Sensitivity analyses

Sensitivity analyses were performed utilizing the same analytic strategy, with alternative case selections or covariates. First, models from the main analyses, including those with co-occurring ADHD, were additionally adjusted for sleep medication use (see above). Second, conservative analyses were conducted based on all participants with an available baseline assessment and imputing 0 (i.e. not fulfilling the response criteria) for all missing data at postintervention and/or 3-month follow-up. Third, given that the original RCTs showed variable effects depending on the duration of the training (ES = 0.32–0.40 in Choque Olsson et al. (2017); ES = 0.76–0.82 in Jonsson et al. (2019)), the main analyses were repeated including only the participants from the larger trial (Choque Olsson et al., 2017).

### Community involvement statement

The trials on which these analyses were based were supported by local and national interest organizations. Furthermore, participant and parent perceptions of the intervention were collected to inform the final development of KONTAKT (Choque Olsson et al., 2016). However, community members were not directly involved in developing the study design, implementation, or interpretation of the findings of the current analyses.

## Results

### Main analyses

#### Reliable improvement

Reliable improvement (⩾25 points) occurred in 30.5% of the participants randomized to KONTAKT, compared with 17.9% of those receiving standard care only, with an aOR of 2.07 (95% CI: 1.09–3.92; NNT = 8). There was no significant three-way (ADHD by age group by study arm) interaction (Supplementary Material, Table S3). However, separate models exploring the underlying two-way interactions suggested study arm (KONTAKT) by ADHD (OR: 0.10, 95% CI: 0.02–0.60) and study arm (KONTAKT) by age group (child) interactions (OR: 0.10, 95% CI: 0.03–0.37; Supplementary Material, Table S4).

**Figure 2.**
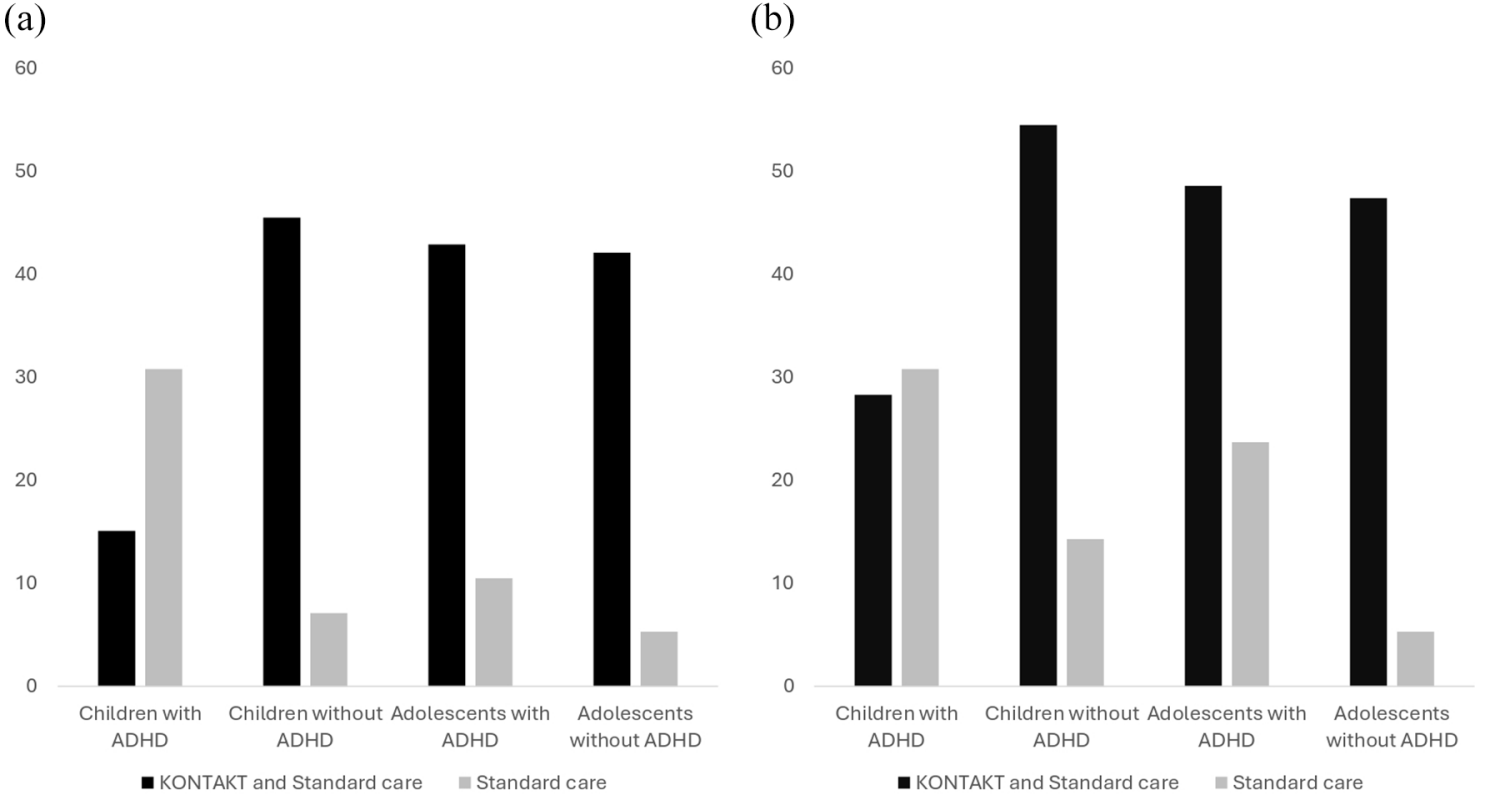
Improvement on the parent-rated Social Responsiveness Scale among autistic children and adolescents with and without co-occurring ADHD in two randomized trials of social skills group training as an add-on to standard care: (a) reliable change (%) and (b) clinically relevant change (%).

Stratifying the sample by co-occurrence of ADHD, there was a significant age group (child) by study arm (KONTAKT) interaction only for the subgroup with ADHD (OR: 0.06, 95% CI: 0.01–0.30). Similarly, stratification based on age group showed a significant interaction effect of ADHD by study group (KONTAKT) among the children (OR: 0.04, 95% CI: 0.003–0.47), but not among the adolescents (Supplementary Material, Table S5). Stratified analyses revealed effects of KONTAKT over and above standard care for adolescents with ADHD and without ADHD, and children without ADHD, but not for children with co-occurring ADHD (see Table 1). Relative to the other groups, there was a notably high response rate from standard care and a low response rate from KONTAKT among the children with ADHD (Figure 2(a)).

**Table 1.** Reliable improvement (⩾25 points on the parent-rated Social Responsiveness Scale) following social skills training or standard care in autistic children and adolescents with or without ADHD.

	Responders			Moderators	Intervention effects	
	KONTAKT™ + standard care (*n* = 118) *n* (%)	Standard care (*n* = 123) *n* (%)	NNT	Interaction effects^a^	OR (95% CI)	Adjusted OR (95% CI)
All	36 (30.5)	22 (17.9)	8	**ADHD ×** **Study arm (*p*** **=** **0.011);** **Age group** × **Study arm (***p* < **0.001);** ADHD × Age group (*p* = 0.787)	**2.02 (1.10–3.69)**	**2.07 (1.09–3.92)** ^b^
With ADHD	23 (26.1)	20 (22.2)	26	**Age group × Study arm (*p*** **<** **0.001)**	1.24 (0.62–2.46)	1.30 (0.63–2.71)^b^
Without ADHD	13 (43.3)	2 (6.1)	3	Age group × Study arm (*p* = 0.909)	**11.85 (2.39–58.82)**	**21.17 (3.14–142.84)** ^b^
Children	13 (20.3)	17 (25.8)	−19	**ADHD × Study arm (*p*** **=** **0.011)**	0.74 (0.32–1.67)	0.84 (0.35–2.04)^b^
With ADHD	8 (15.1)	16 (30.8)	−7	N/A	0.40 (0.15–1.04)	0.43 (0.16–1.20)^c^
Without ADHD	5 (45.5)	1 (7.1)	3	N/A	**10.83 (1.03–114.15)**	**14.18 (1.13–177.97)** ^c^
Adolescents	23 (42.6)	5 (8.8)	3	ADHD × Study arm (*p* = 0.577)	**7.72 (2.66–22.37)**	**7.50 (2.41–23.33)** ^b^
With ADHD	15 (42.9)	4 (10.5)	4	N/A	**6.38 (1.86–21.89)**	**7.29 (1.97–26.91)** ^c^
Without ADHD	8 (42.1)	1 (5.3)	3	N/A	**13.09 (1.44–119.34)**	**24.54 (1.74–347.11)** ^c^

NNT: numbers needed to treat; N/A: not applicable.

a From models including the two-way interaction and underlying main effects.

b Adjusted for preintervention age (years), sex, preintervention score on the parent-rated Social Responsiveness Scale, full-scale IQ score (dichotomized), stimulant use, depression, anxiety disorder, and intervention length.

c Adjusted for preintervention age (years), sex, and preintervention score on the parent-rated Social Responsiveness Scale.

#### Clinically relevant improvement

Clinically relevant improvement in social responsiveness (⩾10 points) was observed in 39.8% of the participants receiving KONTAKT, compared with 22.8% of those receiving standard care only (aOR: 2.28; 95% CI: 1.27–4.11; NNT = 6). There was no significant three-way (ADHD by age group by study arm) interaction (Supplementary Material, Table S3). However, separate models exploring the underlying two-way interaction terms suggested study arm (KONTAKT) by ADHD (OR: 0.15, 95% CI: 0.03–0.68) and age group (child) by study arm (KONTAKT) interactions (OR: 0.30, 95% CI: 0.10–0.94; Supplementary Material, Table S4). When the sample was stratified by co-occurrence of ADHD, the age group by study arm interaction did not reach statistical significance in either group. Stratification based on age group showed a significant interaction effect of ADHD by study group (KONTAKT) among the children (OR: 0.12, 95% CI: 0.02–0.99), but not among the adolescents (Supplementary Material, Table S5). The stratified analyses revealed significant effects of KONTAKT over and above standard care for both adolescents with and without ADHD. Significant effects were also found for children without ADHD, but not for children with co-occurring ADHD (see Table 2). As for reliable improvement, there were relatively high response rates from standard care and a low response rate from KONTAKT among children with ADHD (Figure 2(b)).

**Table 2.** Clinically relevant improvement (⩾10 points on parent-rated Social Responsiveness Scale at postintervention and follow-up) following social skills training or standard care in autistic children and adolescents with or without ADHD.

	Responders			Moderators	Intervention effects	
	KONTAKT™ + standard care (*n* = 118) *n* (%)	Standard care (*n* = 123) *n* (%)	NNT	Interaction effects^a^	OR (95% CI)	Adjusted OR (95% CI)
All	47 (39.8)	28 (22.8)	6	**ADHD × Study arm (*p*** **=** **0.014); Age group × Study arm (*p*** **=** **0.039)**; ADHD × Age group (*p* = 0.396)	**2.25 (1.28–3.93)**	**2.28 (1.27–4.11)** ^b^
With ADHD	32 (36.4)	25 (27.8)	12	Age group × Study arm (*p* = 0.064)	1.49 (0.79–2.80)	1.56 (0.79–3.07)^b^
Without ADHD	15 (50.0)	3 (9.1)	3	Age group × Study arm (*p* = 0.586)	**10.00 (2.50–39.98)**	**18.69 (3.49–99.91)** ^b^
Children	21 (32.8)	18 (27.3)	19	**ADHD × Study arm (*p*** **=** **0.049)**	1.30 (0.61–2.76)	1.58 (0.69–3.63)^b^
With ADHD	15 (28.3)	16 (30.8)	−40	N/A	0.89 (0.38–2.06)	1.14 (0.45–2.89)^c^
Without ADHD	6 (54.5)	2 (14.3)	3	N/A	**7.20 (1.07–48.64)**	**19.64 (1.55–248.66)** ^c^
Adolescents	26 (48.1)	10 (17.5)	4	ADHD × Study arm (*p* = 0.176)	**4.36 (1.84–10.38)**	**4.21 (1.65–10.75)** ^b^
With ADHD	17 (48.6)	9 (23.7)	5	N/A	**3.04 (1.12–8.27)**	**3.41 (1.18–9.82)** ^c^
Without ADHD	9 (47.4)	1 (5.3)	3	N/A	**16.20 (1.79–147.07)**	**17.73 (1.74–181.08)** ^c^

NNT: numbers needed to treat; N/A: not applicable.

a From models including the two-way interaction term and underlying main effects.

b Adjusted for preintervention age (years), sex, preintervention score on the parent-rated Social Responsiveness Scale, full-scale IQ score (dichotomized), stimulant use, depression, anxiety disorder, and intervention length.

c Adjusted for preintervention age (years), sex, and preintervention score on the parent-rated Social Responsiveness Scale.

### Sensitivity analyses

Additional adjustments for sleep medication in models including those with co-occurring ADHD did not change estimates materially (data not shown). In the more conservative sensitivity analyses, including all participants providing data preintervention, the estimates were somewhat attenuated; some of the effects observed in the main analyses did not reach statistical significance. Still, the overall pattern of results remained unchanged. The same was true for the sensitivity analyses using data only from the larger trial (see Supplementary Material, Tables S6 to S9).

## Discussion

This study explored whether response to SSGT in autistic children and adolescents was moderated by co-occurring ADHD. Using two distinct response criteria, we found that ADHD did moderate the effect of the KONTAKT program as an add-on to standard care in child and adolescent mental health services. Explorative analyses further suggested that preadolescent participants with ADHD, in particular, seemed to gain little from the training. These findings add nuance to previous results suggesting that adolescents have a more favorable outcome of KONTAKT than children (Choque Olsson et al., 2017).

While social functioning can be problematic both for youth with autism and ADHD (Petrina et al., 2014; Ros & Graziano, 2018), children with ADHD may experience greater performance-related challenges (Mikami et al., 2017). Efforts to specifically target the enactment of social skills in autistic youths with ADHD have so far not resulted in intended outcomes (Gerber et al., 2022), underscoring the need for further improvement of the intervention-to-person fit (Gates et al., 2023). In interpreting our results, the fact that all participants without ADHD had co-occurring depression or anxiety disorder should also be considered. It has previously been reported that participants with co-occurring anxiety can benefit from SSGT (e.g. Factor et al., 2022), possibly due to overlapping intervention strategies to support both social skills and alleviate anxiety symptoms (Afsharnejad et al., 2021). The age-dependent effects of co-occurring ADHD explored in this study also align with findings from adjacent literature. For instance, Guo et al. (2022) suggest that increasing age is related to greater gains from psychosocial interventions, including CBT, for populations with ADHD. Age-related changes in symptom manifestation of ADHD may be a contributing factor (Hartman et al., 2016). For instance, adolescents may be able to assimilate the intervention despite experiencing inner restlessness or occasionally losing track during sessions.

In contrast to previous research on SSGT, which mainly has reported mean differences and effect sizes on a group level, this study focused on response at an individual level. Such information is crucial to enable clinicians and service users to make informed decisions. Our findings suggest that reliable improvement in one additional patient (compared to standard care alone) on average requires that eight patients receive KONTAKT (NNT = 8). Similarly, approximately six patients, on average, must receive the intervention to achieve clinically relevant improvement in one additional patient (NNT = 6). Estimates varied substantially between subgroups, with NNTs of three in those without ADHD, four to five in adolescents with co-occurring ADHD, and a negative NNT for children with co-occurring ADHD. Overall, the estimated NNT is comparable to what previously has been reported for psychosocial interventions in child and adolescent mental health services, including CBT for common mental health conditions (James et al., 2020; Jeppesen et al., 2021; Viswanathan et al., 2020).

### Clinical implications

Some recent studies may provide some clues on possible strategies to improve the outcome for children with co-occurring ADHD. For instance, Dekker et al. (2021) found that children with high levels of hyperactive/impulsive symptoms seemed to improve only when receiving more wrap-around support from parents and teachers. Furthermore, Harkins and Mazurek (2024) used age-adapted program protocols, adding developmentally appropriate content and hands-on activities rather than whole group discussion-based learning for elementary-aged participants, and found that co-occurring ADHD did not impact the outcome. Similarly, an extended version of the PEERS program, using structured social skills training with less focus on theoretical discussion and involving parents as social coaches every session, resulted in similar gains for autistic adolescents with and without co-occurring ADHD (Geannopoulos et al., 2024). This tentatively suggests that increased involvement of parents as co-teachers, less theoretical discussion, and more hands-on practice activities are promising strategies to help children with ADHD gain more from SSGT.

### Limitations

Our findings should be interpreted with some limitations in mind. First, while the overall study sample was relatively large, the original trials were not designed for moderation analyses. Notably, the three-way interaction failed to confirm that the moderating effect of ADHD differed between children and adolescents, possibly due to a lack of statistical power. Therefore, the exploratory stratified analyses should be interpreted with caution and require independent replication. In addition, the precision of some of the estimates from the stratified analyses suffered from imbalanced group sizes, with 74% of the analytic sample having co-occurring ADHD. This imbalance presumably reflects the high prevalence of ADHD in this population (e.g. Hossain et al., 2020) and the clinical setting of the study. Second, while the two distinct definitions of response used here led to a similar pattern of results in both the main and sensitivity analyses, we cannot rule out that other definitions of response, using other cut-offs or alternative outcome measures, would present a somewhat different picture. Secondary outcomes from the original studies were not included in this study, and previous research suggests that autistic children with ADHD may experience gains not captured by the SRS (Gerber et al., 2022). It should also be noted that no less than 22%–28% (depending on criteria) of those with co-occurring ADHD who were assigned standard care were classified as responders, compared to 6%–9% of those without ADHD, indicating that measurements might work differently depending on age and presence of ADHD. Third, it is unclear to what extent the pattern of results in this study generalizes to other SSGT programs and service settings. Importantly, it is not known how KONTAKT interacts with standard care. The value added by KONTAKT may partly depend on what other treatments and support are available to the service user. For instance, the gains from SSGT may be overshadowed by the immediate effects of pharmacological treatment in children with ADHD. In addition, standard care may vary depending on service settings and over time. Finally, the ADHD diagnoses obtained from medical records were not corroborated by the research team. In addition, the original trials were initiated at a time when diagnostic manuals formally did not permit the co-occurrence of autism and ADHD. ADHD may therefore have been underdiagnosed. However, this should have been the exception rather than the rule, since clinical practice at the time was already aligned with the upcoming change to the diagnostic manuals. Overall, these limitations underscore the preliminary and exploratory nature of our findings. Replication in other contexts and samples is needed, which is further underscored by recent research pointing to the elusive nature of reliable predictors of treatment outcomes in psychiatric research (Chekroud et al., 2024). Future research should also ensure community involvement and co-production throughout the research process.

## Conclusion

This study tentatively suggests that co-occurring ADHD may affect the outcome of SSGT, especially in preadolescent children. Awaiting replication of this finding and relevant adjustments to the intervention protocol, group leaders should carefully monitor the progress of this subgroup of participants and provide additional support as necessary. Overall, our findings highlight that clinical characteristics and stages of development may partially explain individual differences in response to SSGT. Future research should explore this further, to enable more personalized services for autistic children and adolescents.

## Supplemental Material

sj-docx-1-aut-10.1177_13623613251331993 – Supplemental material for The moderating role of co-occurring attention-deficit hyperactivity disorder in social skills group training for autistic children and adolescents Supplemental material, sj-docx-1-aut-10.1177_13623613251331993 for The moderating role of co-occurring attention-deficit hyperactivity disorder in social skills group training for autistic children and adolescents by Anna Fridell, Nora Choque Olsson, Christina Coco, Sven Bölte and Ulf Jonsson in Autism
