# Supplementary material for: The moderating role of co-occurring attention-deficit hyperactivity disorder in social skills group training for autistic children and adolescents
Source: Autism. 2025 Apr 23;29(8):2030–43. doi: 10.1177/13623613251331993 (PMC12255842; doi:10.1177/13623613251331993)
Supplement: sj-docx-1-aut-10.1177_13623613251331993 – Supplemental material for The moderating role of co-occurring attention-deficit hyperactivity disorder in social skills group training for autistic children and adolescents [file sj-docx-1-aut-10.1177_13623613251331993.docx]

**Supplementary material**

*The moderating role of ADHD in social skills group training for autistic children and adolescents*

Content

[**Table S1.** Demographic and clinical characteristics. 2](#_Toc183352455)

[**Table S2**: Demographic and clinical characteristics of completers to non-completers. 5](#_Toc183352456)

[**Table S3.** Logistic regression models testing the interaction of study arm, ADHD, and age group, including the three-way interaction term and all underlying two-way interaction terms and main effects. 7](#_Toc183352457)

[**Table S4.** Logistic regression models testing two-way interactions in the full sample. 8](#_Toc183352458)

[**Table S5.** Explorative logistic regression models testing two-way interactions in the samples split by co-occurring ADHD and age group. 9](#_Toc183352459)

[**Table S6.** Reliable improvement (≥25 points on parent-rated Social Responsiveness Scale) following social skills training or standard care in autistic children and adolescents with or without ADHD – **using all available data** and imputing 0 (not improved) for missing data. 10](#_Toc183352460)

[**Table S7.** Clinically relevant improvement (≥10 points on parent-rated Social Responsiveness Scale at both postintervention and follow-up) following social skills training or standard care in autistic children and adolescents with or without ADHD – **using all available data** and imputing 0 (not improved) for missing data. 11](#_Toc183352461)

[**Table S8.** Reliable improvement (≥25 points on parent-rated Social Responsiveness Scale) following social skills training or standard care in autistic children and adolescents with or without ADHD – using complete cases from **the larger trial only.** 12](#_Toc183352462)

[**Table S9.** Clinically relevant improvement (≥10 points on parent-rated Social Responsiveness Scale at both postintervention and follow-up) following social skills training or standard care in autistic children and adolescents with or without ADHD – using complete cases from **the larger trial only.** 13](#_Toc183352463)

## **Table S1.** Demographic and clinical characteristics.

|  | **Total**  **(N=241)** | | | **Children**  **(n=130)** | | | **Adolescents**  **(n=111)** | | |
| --- | --- | --- | --- | --- | --- | --- | --- | --- | --- |
|  | **With**  **ADHD**  **(n=178)** | **Without**  **ADHD**  **(n=63)** |  | **With**  **ADHD**  **(n=105)** | **Without**  **ADHD**  **(n=25)** |  | **With ADHD**  **(n=73)** | **Without**  **ADHD**  **(n=38)** |  |
|  | ***Mean***  ***(SD)*** | ***Mean (SD)*** |  | ***Mean (SD)*** | ***Mean (SD)*** |  | ***Mean (SD)*** | ***Mean (SD)*** |  |
| Age  (years) | 12 (3) | 13 (3) | ***p*=.005** | 10 (2) | 10 (1) | *p*=.679 | 14 (1) | 15 (1) | *p*=.237 |
| ADOS  (total score) | 11 (4) | 11 (3) | *p*=.572 | 11 (3) | 11 (3) | *p*=.938 | 11 (4) | 10 (3) | *p*=.559^a^ |
| Parental age (years) | 46 (5) | 48 (7) | ***p*=.003** | 45 (5) | 46 (7) | *p*=.468 | 47 (5) | 50 (6) | ***p*=.008** |
| Parental education (years) | 14 (3) | 15 (3) | *p*=.084 | 14 (3) | 16 (2) | *p*=.061 | 14 (3) | 15 (3) | *p*=.467 |
| SRS  (pre-intervention) | 87 (24) | 83 (23) | *p*=.255 | 86 (23) | 88 (18) | *p*=.650 | 89 (25) | 80 (26) | *p*=.076 |
|  | ***Count***  ***n (%)*** | ***Count***  ***n (%)*** |  | ***Count***  ***n (%)*** | ***Count***  ***n (%)*** |  | ***Count***  ***n (%)*** | ***Count***  ***n (%)*** |  |
| Sex  (male) | 129 (72.5) | 42 (66.7) | *p*=.383 | 80 (76.2) | 19 (76.0) | *p*=.984 | 49 (67.1) | 23 (60.5) | *p*=.490 |
| Study arm  (KONTAKT^TM^) | 88 (49.4) | 30 (47.6) | *p*=.804 | 53 (50.5) | 11 (44.0) | *p*=.561 | 35 (47.9) | 19 (50.0) | *p*=.837 |
| Intervention length  (12 weeks) | 152 (85.4) | 50 (79.4) | *p*=.264 | 96 (91.4) | 20 (80.0) | *p*=.143^b^ | 56 (76.7) | 30 (78.9) | *p*=.789 |
| Native language  (Swedish) | 162 (91.0) | 54 (85.7) | *p*=.236 | 96 (91.4) | 20 (80.0) | *p*=.143^b^ | 66 (90.4) | 34 (89.5) | *p*=1.000^b^ |
| WISC score  (≥85) |  |  |  |  |  |  |  |  |  |
| Full-scale IQ | 144 (80.9) | 58 (92.1) | ***p*=.039** | 85 (81.0) | 22 (88.0) | *p*=.564^b^ | 59 (80.8) | 36 (94.7) | ***p*=.048** |
| Performance | 162 (91.0) | 60 (95.2) | *p*=.416^b^ | 97 (92.4) | 24 (96.0) | *p*=1.000^b^ | 65 (89.0) | 36 (94.7) | *p*=.490^b^ |
| Verbal | 147 (82.6) | 54 (85.7) | *p*=.566 | 85 (81.0) | 19 (76.0) | *p*=.578 | 62 (84.9) | 35 (92.1) | *p*=.374^b^ |
| Working memory | 113 (63.5) | 53 (85.5) | ***p*=.001** | 68 (64.8) | 22 (88.0) | ***p*=.024** | 45 (61.6) | 31 (83.8) | ***p*=.018** |
| Processing speed | 109 (61.2) | 43 (69.4) | *p*=.253 | 66 (62.9) | 17 (68.0) | *p*=.631 | 43 (58.9) | 26 (70.3) | *p*=.244 |
| Pharmacological treatment |  |  |  |  |  |  |  |  |  |
| Stimulants | 116 (65.2) | 2 (3.2) | ***p*<.001** | 67 (63.8) | 1 (4.0) | ***p*<.001** | 49 (67.1) | 1 (2.6) | ***p*<.001** |
| Sleep inducing | 36 (20.2) | 5 (7.9) | ***p*=.026** | 18 (17.1) | 2 (8.0) | *p*=.361^b^ | 18 (24.7) | 3 (7.9) | ***p*=.032** |
| SSRIs | 28 (15.7) | 13 (20.6) | *p*=.373 | 8 (7.6) | 4 (16.0) | *p*=.244^b^ | 20 (27.4) | 9 (23.7) | *p*=.673 |
| Individual psychological treatment |  |  |  |  |  |  |  |  |  |
| CBT | 2 (1.1) | 4 (6.3) | ***p*=.042^b^** | 2 (1.9) | 1 (4.0) | *p*=.476^b^ | 0 (0) | 3 (7.9) | ***p*=.038^b^** |
| General counselling | 12 (6.7) | 7 (11.1) | *p*=.282^b^ | 3 (2.9) | 2 (8.0) | *p*=.245^b^ | 9 (12.3) | 5 (13.2) | *p*=1.000^b^ |
| Habilitation services |  |  |  |  |  |  |  |  |  |
| Parental psycho-education | 44 (24.7) | 17 (27.0) | *p*=.722 | 31 (29.5) | 5 (20.0) | *p*=.339 | 13 (17.8) | 12 (31.6) | *p*=.099 |
| Other | 9 (5.1) | 4 (6.3) | *p*=.747^b^ | 3 (4.1) | 3 (7.9) | *p*=1.000^b^ | 6 (5.7) | 1 (4.0) | *p*=.410^b^ |
| Mental health conditions |  |  |  |  |  |  |  |  |  |
| Depression | 8 (4.5) | 25 (39.7) | ***p*<.001** | 3 (2.9) | 6 (24.0) | ***p*<.001** | 5 (6.8) | 19 (50) | ***p*<.001** |
| Anxiety disorder | 9 (5.1) | 48 (76.2) | ***p*<.001** | 1 (1.0) | 21 (84.0) | ***p*<.001** | 8 (11.0) | 27 (71.1) | ***p*<.001** |
| Other^c^ | 25 (14.0) | 7 (11.1) | *p*=.555 | 19 (18.1) | 3 (12.0) | *p*=.465 | 6 (8.2) | 4 (10.5) | *p*=.687 |

ADOS: Autism Diagnostic Observation Schedule; CBT: Cognitive Behavioural Therapy; IQ: Intellectual Quotient; SRS: Social Responsiveness Scale; SSRI: Selective Serotonin Reuptake Inhibitor; WISC: Wechsler Intelligence Scale for Children.

^a^Equal variances not assumed

^b^Fisher’s Exact

^c^e.g., obsessive-compulsive disorder

## **Table S2**: Demographic and clinical characteristics of completers to non-completers.

|  | **Total**  **(N=346)** | | |
| --- | --- | --- | --- |
|  | **Completers**  **(n=241)** | **Non-completers**  **(n=105)** |  |
|  | ***Mean (SD)*** | ***Mean (SD)*** |  |
| Age  (years) | 12 (3) | 12 (3) | *p*=.620 |
| ADOS  (total score) | 11 (3) | 11 (4) | *p*=.383 |
| Parental age  (years) | 46 (6) | 46 (6) | *p*=.236 |
| Parental education  (years) | 15 (3) | 14 (3) | *p*=.184 |
| SRS  (preintervention) | 86 (24) | 94 (26) | ***p*=.008** |
|  | ***Count***  ***n (%)*** | ***Count***  ***n (%)*** |  |
| Age group  (children) | 130 (53.9) | 60 (57.1) | *p*=.582 |
| Sex  (male) | 171 (71.0) | 72 (68.6) | *p*=.656 |
| Study arm  (KONTAKT^TM^) | 118 (49.0) | 55 (52.4) | *p*=.559 |
| Intervention length  (12 weeks) | 202 (83.8) | 94 (89.5) | *p*=.165 |
| Native language  (Swedish) | 216 (89.6) | 99 (94.3) | *p*=.163 |
| ADHD | 178 (73.9) | 84 (80.0) | *p*=.221 |
| WISC score  (≥85) |  |  |  |
| Full scale IQ | 202 (83.8) | 77 (73.3) | ***p*=.023** |
| Performance | 222 (92.1) | 102 (97.1) | *p*=.078 |
| Verbal | 201 (83.4) | 83 (79.0) | *p*=.332 |
| Working memory | 166 (69.2) | 58 (55.8) | ***p*=.017** |
| Processing speed | 152 (63.3) | 57 (54.8) | *p*=.137 |
| Pharmacological treatment |  |  |  |
| Central stimulants | 118 (49.0) | 59 (56.2) | *p*=.216 |
| Sleep inducing | 41 (17.0) | 32 (30.5) | ***p*=.005** |
| SSRIs | 41 (17.0) | 22 (21.0) | *p*=.383 |
| Individual psychological treatment |  |  |  |
| CBT | 6 (2.5) | 5 (4.8) | *p*=.319^a^ |
| General counselling | 19 (7.9) | 5 (4.8) | *p*=.293 |
| Habilitation services |  |  |  |
| Parental psychoeducation | 61 (25.3) | 20 (19.0) | *p*=.206 |
| Other | 13 (5.4) | 3 (2.9) | *p*=.409^a^ |
| Mental health conditions |  |  |  |
| Depression | 33 (13.7) | 16 (15.2) | *p*=.705 |
| Anxiety disorder | 57 (23.7) | 17 (16.2) | *p*=.120 |
| Other^b^ | 32 (13.3) | 19 (18.1) | *p*=.245 |

ADOS: Autism Diagnostic Observation Schedule, CBT: Cognitive Behavioural Therapy; IQ: Intellectual Quotient, SRS: Social Responsiveness Scale, SSRI: Selective Serotonin Reuptake Inhibitor, WISC: Wechsler Intelligence Scale for Children.

^a^Fisher’s Exact

^b^e.g., obsessive-compulsive disorder

## **Table S3.** Logistic regression models testing the interaction of study arm, ADHD, and age group, including the three-way interaction term and all underlying two-way interaction terms and main effects.

|  | **Reliable improvement** | | **Clinically relevant improvement** | |
| --- | --- | --- | --- | --- |
|  | **OR (95% CI)** | **p** | **OR (95% CI)** | **p** |
| ADHD(yes)*Age group(child)*Study arm(KONTAKT) | 0.76 (0.00-2.74) | .159 | 0.66 (0.03-16.05) | .796 |
| ADHD(yes)*Age group(child) | 2.73 (0.12-60.58) | .526 | 0.48 (0.03-6.99) | .589 |
| ADHD(yes)*Study arm(KONTAKT) | 0.49 (0.04-6.12) | .577 | 0.19 (0.02-2.12) | .176 |
| Age group(child)*Study arm(KONTAKT) | 0.83 (0.03-20.91) | .909 | 0.44 (0.02-8.23) | .586 |
| ADHD(yes) | 2.12 (0.22-20.39) | .516 | 5.59 (0.65-47.86) | .232 |
| Study arm(KONTAKT) | **13.09 (1.44-119.44)** | **.023** | **16.20 (1.79-147.07)** | **.0.78** |
| Age group(child) | 1.39 (0.08-24.23) | .824 | 3.00 (0.24-36.88) | .391 |

## **Table S4.** Logistic regression models testing two-way interactions in the full sample.

|  | **Reliable improvement** | | **Clinically relevant improvement** | |
| --- | --- | --- | --- | --- |
|  | **OR (95% CI)** | **p** | **OR (95% CI)** | **p** |
| **Model 1** |  |  |  |  |
| ADHD(yes)*Study arm(KONTAKT) | **0.10 (0.02-0.60)** | **.011** | **0.15 (0.03-0.68)** | **.014** |
| ADHD(yes) | 4.43 (0.98-20.12) | .054 | **3.85 (1.08-13.74)** | **.038** |
| Study arm(KONTAKT) | **11.85 (2.39-58.52)** | **.002** | **10.00 (2.50-39.98)** | **.001** |
| **Model 2** |  |  |  |  |
| Age group(child)*Study arm(KONTAKT) | **0.10 (0.03-0.37)** | **<.001** | **0.30 (0.10-0.94)** | **.039** |
| Age group(child) | **3.61 (1.24-10.53)** | **.019** | 1.76 (0.74-4.21) | .202 |
| Study arm(KONTAKT) | **7.72 (2.66-22.37)** | **<.001** | **4.36 (1.84-10.38)** | **<.001** |
| **Model 3** |  |  |  |  |
| ADHD(yes)*Age group(child) | 0.83 (0.21-3.26) | .787 | 0.58 (0.16-2.06) | .396 |
| ADHD(yes) | 1.13 (0.46-2.82) | .787 | 1.55 (0.65-3.68) | .322 |
| Age group(child) | 1.02 (0.31-3.33) | .977 | 1.32 (0.44-3.99) | .626 |

## **Table S5.** Explorative logistic regression models testing two-way interactions in the samples split by co-occurring ADHD and age group.

|  | **Reliable improvement** | | **Clinically relevant improvement** | |
| --- | --- | --- | --- | --- |
|  | **OR (95% CI)** | **p** | **OR (95% CI)** | **p** |
| **With ADHD** |  |  |  |  |
| Age group(child)*Study arm(KONTAKT) | **0.06 (0.01-0.30)** | **<.001** | 0.29 (0.08-1.08) | .064 |
| Age group(child) | **3.78 (1.15-12.44)** | **.029** | 1.43 (0.55-3.71) | .460 |
| Study arm(KONTAKT) | **6.38 (1.86-21.89)** | **.003** | **3.04 (1-12-8.27)** | **.029** |
| **Without ADHD** |  |  |  |  |
| Age group(child)*Study arm(KONTAKT) | 0.83 (0.03-20.91) | .909 | 0.44 (0.02-8.23) | .586 |
| Age group(child) | 0.82 (0.08-24.23) | .824 | 3.00 (0.24-36.88) | .391 |
| Study arm(KONTAKT) | **13.09 (1.44-119.34)** | **.023** | **16.20 (01.79-147.07)** | **.013** |
| **Children** |  |  |  |  |
| ADHD(yes)*Study arm(KONTAKT) | **0.04 (0.00-0.47)** | **.011** | **0.12 (0.02-0.99)** | **.049** |
| ADHD(yes) | 5.78 (0.70-48.02) | .104 | 2.67 (0.53-13.32) | .232 |
| Study arm(KONTAKT) | **10.83 (1.03-114.15)** | **.047** | **7.20 (1.07-48.64)** | **.043** |
| **Adolescents** |  |  |  |  |
| ADHD(yes)*Study arm(KONTAKT) | 0.49 (0.04-6.12) | .577 | 0.19 (0.02-2.11) | .176 |
| ADHD(yes) | 2.12 (0.22-20.39) | .516 | 5.59 (0.65-47.86) | .116 |
| Study arm(KONTAKT) | **13.09 (1.43-119.34)** | **.023** | **16.20 (1.79-147.07)** | **.013** |

## **Table S6.** Reliable improvement (≥25 points on parent-rated Social Responsiveness Scale) following social skills training or standard care in autistic children and adolescents with or without ADHD – **using all available data** and imputing 0 (not improved) for missing data.

|  | **Response** | | **Moderators** | **Intervention effects** | |
| --- | --- | --- | --- | --- | --- |
|  | **KONTAKT**^TM^ **+ Standard care**  **(n=173)**  **n (%)** | **Standard care**  **(n=173)**  **n (%)** | **Significant interaction effects^a^** | **OR**  **(95% CI)** | **Adjusted OR**  **(95% CI)** |
| All | 37 (21.4) | 26 (15.0) | **ADHD*Study arm;**  **Age group*Study arm** | 1.54  (0.89-2.67) | 1.49  (0.84-2.63)^b^ |
| With ADHD | 23 (17.6) | 24 (18.3) | **Age group*study arm** | 0.95  (0.51-1.79) | 0.90  (0.47-1.73)^b^ |
| Without ADHD | 14 (33.3) | 2 (4.8) | Not significant | **10.00**  **(2.11-47.51)** | **15.22**  **(2.72-85.08)**^b^ |
| Children | 13 (14.3) | 20 (20.2) | **ADHD*study arm** | 0.66  (0.31-1.42) | 0.68  (0.31-1.49)^b^ |
| With ADHD | 8 (10.4) | 19 (23.8) | N/A | **0.37**  **(0.15-0.91)** | **0.38**  **(0.15-0.95)**^c^ |
| Without ADHD | 5 (35.7) | 1 (5.3) | N/A | **10.00**  **(1.01-98.88)** | **15.96**  **(1.28-198.49)**^c^ |
| Adolescents | 24 (29.3) | 6 (8.1) | Not significant | **4.69**  **(1.79-12.26)** | **3.97**  **(1.45-10.86)**^b^ |
| With ADHD | 15 (27.8) | 5 (9.8) | N/A | **3.54**  **(1.18-10.61)** | **3.29**  **(1.07-10.08)^c^** |
| Without ADHD | 9 (32.1) | 1 (4.3) | N/A | **10.42**  **(1.21-89.93)** | **12.21**  **(1.22-122.38)**^c^ |

^a^From models including the two-way interaction term and underlying main effects.

^b^Adjusted for preintervention age (years), sex, preintervention score on the parent-rated Social Responsiveness Scale, full-scale IQ score (dichotomized), stimulant use, depression, anxiety disorder, and intervention length.

^c^Adjusted for preintervention age (years), sex, and preintervention score on the parent-rated Social Responsiveness Scale.

## **Table S7.** Clinically relevant improvement (≥10 points on parent-rated Social Responsiveness Scale at both postintervention and follow-up) following social skills training or standard care in autistic children and adolescents with or without ADHD – **using all available data** and imputing 0 (not improved) for missing data.

|  | **Response** | | **Moderators** | **Intervention effects** | |
| --- | --- | --- | --- | --- | --- |
|  | **KONTAKT**^TM^ **+ Standard care**  **(n=173)**  **n (%)** | **Standard care**  **(n=173)**  **n (%)** | **Significant interaction effects^a^** | **OR**  **(95% CI)** | **Adjusted OR**  **(95% CI)** |
| All | 47 (27.2) | 28 (16.2) | **ADHD*Study arm** | **1.93**  **(1.14-3.27)** | **1.92**  **(1.12-3.27)**^b^ |
| With ADHD | 32 (24.4) | 25 (19.1) | Not significant | 1.37  (0.76-2.47) | 1.32  (0.72-2.43)^b^ |
| Without ADHD | 15 (35.7) | 3 (7.1) | Not significant | **7.22**  **(1.90-27.39)** | **15.99**  **(3.24-78.84)**^b^ |
| Children | 21 (23.1) | 18 (18.2) | Not significant | 1.35  (0.67-2.74) | 1.44  (0.69-2.99)^b^ |
| With ADHD | 15 (19.5) | 16 (20.0) | N/A | 0.97  (0.44-2.12) | 1.01  (0.45-2.26)^c^ |
| Without ADHD | 6 (42.9) | 2 (10.5) | N/A | **6.38**  **(1.05-38.86)** | **21.47**  **(1.80-256.49)**^c^ |
| Adolescents | 26 (31.7) | 10 (13.5) | Not significant | **2.97**  **(1.32-6.70)** | **2.89**  **(1.22-6.84)**^b^ |
| With ADHD | 17 (31.5) | 9 (17.6) | N/A | 2.14  (0.85-5.39) | 2.04  (0.79-5.23)^c^ |
| Without ADHD | 9 (32.1) | 1 (4.3) | N/A | **10.42**  **(1.21-89.93)** | **12.05**  **(1.29-112.72)**^c^ |

^a^From models including the two-way interaction term and underlying main effects.

^b^Adjusted for preintervention age (years), sex, preintervention score on the parent-rated Social Responsiveness Scale, full-scale IQ score (dichotomized), stimulant use, depression, anxiety disorder, and intervention length.

^c^Adjusted for preintervention age (years), sex, and preintervention score on the parent-rated Social Responsiveness Scale.

## **Table S8.** Reliable improvement (≥25 points on parent-rated Social Responsiveness Scale) following social skills training or standard care in autistic children and adolescents with or without ADHD – using complete cases from **the larger trial only.**

|  | **Response** | | **Moderators** | **Intervention effects** | |
| --- | --- | --- | --- | --- | --- |
|  | **KONTAKT**^TM^ **+ Standard care**  **(n=102)**  **n (%)** | **Standard care**  **(n=100)**  **n (%)** | **Significant interaction effects^a^** | **OR**  **(95% CI)** | **Adjusted OR**  **(95% CI)** |
| All | 29 (28.4) | 21 (21.0) | **ADHD*Study arm;**  **Age group*Study arm** | 1.49  (0.78-2.85) | 1.49  (0.76-2.93)^b^ |
| With ADHD | 20 (26.0) | 19 (25.3) | **Age group*Study arm** | 1.03  (0.50-2.14) | 1.03  (0.47-2.23)^b^ |
| Without ADHD | 9 (36.0) | 2 (8.0) | Not significant | **6.47**  **(1.23-34.01)** | **7.87**  **(1.21-51.27)**^b^ |
| Children | 11 (18.6) | 16 (28.1) | **ADHD*Study arm** | 0.59  (0.25-1.41) | 0.64  (0.25-1.63)^b^ |
| With ADHD | 7 (14.3) | 15 (31.9) | N/A | **0.36**  **(0.13-0.97)** | 0.36  (0.12-1.05)^c^ |
| Without ADHD | 4 (40.0) | 1 (10.0) | N/A | 6.00  (0.53-67.65) | 7.84  (0.58-106.58)^c^ |
| Adolescents | 18 (41.9) | 5 (11.6) | Not significant | **5.47**  **(1.80-16.64)** | **4.94**  **(1.53-15.93)**^b^ |
| With ADHD | 13 (46.4) | 4 (14.3) | N/A | **5.20**  **(1.43-18.95)** | **5.90**  **(1.51-22.98)**^c^ |
| Without ADHD | 5 (33.3) | 1 (6.7) | N/A | 7.00  (0.71-69.49) | 13.57  (0.78-246.15)^c^ |

^a^From models including the two-way interaction term and underlying main effects.

^b^Adjusted for preintervention age (years), sex, preintervention score on the parent-rated Social Responsiveness Scale, full-scale IQ score (dichotomized), stimulant use, depression, and anxiety disorder.

^c^Adjusted for preintervention age (years), sex, and preintervention score on the parent-rated Social Responsiveness Scale.

## **Table S9.** Clinically relevant improvement (≥10 points on parent-rated Social Responsiveness Scale at both postintervention and follow-up) following social skills training or standard care in autistic children and adolescents with or without ADHD – using complete cases from **the larger trial only.**

|  | **Response** | | **Moderators** | **Intervention effects** | |
| --- | --- | --- | --- | --- | --- |
|  | **KONTAKT**^TM^ **+ Standard care**  **(n=102)**  **n (%)** | **Standard care**  **(n=100)**  **n (%)** | **Significant interaction effects^a^** | **OR**  **(95% CI)** | **Adjusted OR**  **(95% CI)** |
| All | 40 (39.2) | 26 (26.0) | **ADHD*Study arm;**  **Age group*Study arm** | **1.84**  **(1.01-3.34)** | **1.89**  **(1.01-3.55)**^b^ |
| With ADHD | 28 (36.4) | 24 (32.0) | Not significant | 1.21  (0.62-2.38) | 1.27  (0.62-2.61)^b^ |
| Without ADHD | 12 (48.0) | 2 (8.0) | Not calculated^d^ | **10.62**  **(2.05-54.95)** | **29.20**  **(2.89-295.25)**^b^ |
| Children | 19 (32.2) | 18 (31.6) | Not significant | 1.03  (0.47-2.25) | 1.28  (0.55-3.00)^b^ |
| With ADHD | 14 (28.6) | 16 (34.0) | N/A | 0.78  (0.33-1.84) | 0.99  (0.39-2.55)^c^ |
| Without ADHD | 5 (50.0) | 2 (20.0) | N/A | 4.00  (0.55-29.10) | 11.23  (0.86-146.70)^c^ |
| Adolescents | 21 (48.8) | 8 (18.6) | Not calculated^d^ | **4.18**  **(1.58-11.05)** | **3.82**  **(1.30-11.20)**^b^ |
| With ADHD | 14 (50.0) | 8 (28.6) | N/A | 2.50  (0.83-7.55) | 2.78  (0.88-8.84)^c^ |
| Without ADHD | 7 (46.7) | 0 (0.0) | N/A | Not calculated^d^ | Not calculated^d^ |

^a^From models including the two-way interaction term and underlying main effects.

^b^Adjusted for preintervention age (years), sex, preintervention score on the parent-rated Social Responsiveness Scale, full-scale IQ score (dichotomized), stimulant use, depression, and anxiety disorder.

^c^Adjusted for preintervention age (years), sex, and preintervention score on the parent-rated Social Responsiveness Scale.

^d^Not calculated; zero events in standard care for autistic adolescents without ADHD precluded calculations involving this outcome.
